# The Astonishing Diversity of Ig Classes and B Cell Repertoires in Teleost Fish

**DOI:** 10.3389/fimmu.2013.00028

**Published:** 2013-02-13

**Authors:** Simon Fillatreau, Adrien Six, Susanna Magadan, Rosario Castro, J. Oriol Sunyer, Pierre Boudinot

**Affiliations:** ^1^Deutsches Rheuma-Forschungszentrum, Leibniz InstituteBerlin, Germany; ^2^UPMC Univ Paris 06, UMR 7211, “Immunology, Immunopathology, Immunotherapy”F-75013 Paris, France; ^3^UMR 7211, “Immunology, Immunopathology, Immunotherapy,” CNRSParis, France; ^4^Virologie et Immunologie Moléculaires, Institut National de la Recherche AgronomiqueJouy-en-Josas, France; ^5^Department of Pathobiology, School of Veterinary Medicine, University of PennsylvaniaPhiladelphia, PA, USA

**Keywords:** fish, antibody, repertoire, evolution, B cells

## Abstract

With lymphoid tissue anatomy different than mammals, and diverse adaptations to all aquatic environments, fish constitute a fascinating group of vertebrate to study the biology of B cell repertoires in a comparative perspective. Fish B lymphocytes express immunoglobulin (Ig) on their surface and secrete antigen-specific antibodies in response to immune challenges. Three antibody classes have been identified in fish, namely IgM, IgD, and IgT, while IgG, IgA, and IgE are absent. IgM and IgD have been found in all fish species analyzed, and thus seem to be primordial antibody classes. IgM and IgD are normally co-expressed from the same mRNA through alternative splicing, as in mammals. Tetrameric IgM is the main antibody class found in serum. Some species of fish also have IgT, which seems to exist only in fish and is specialized in mucosal immunity. IgM/IgD and IgT are expressed by two different sub-populations of B cells. The tools available to investigate B cell responses at the cellular level in fish are limited, but the progress of fish genomics has started to unravel a rich diversity of IgH and immunoglobulin light chain locus organization, which might be related to the succession of genome remodelings that occurred during fish evolution. Moreover, the development of deep sequencing techniques has allowed the investigation of the global features of the expressed fish B cell repertoires in zebrafish and rainbow trout, in steady state or after infection. This review provides a description of the organization of fish Ig loci, with a particular emphasis on their heterogeneity between species, and presents recent data on the structure of the expressed Ig repertoire in healthy and infected fish.

## Introduction

Teleost fish form a large zoological group with about 40,000 identified species, in comparison to 10,000 species for birds, and only around 5700 species for mammals. Fish are heterogeneous with regards to size, morphology, physiology, and behavior. They are ubiquitous throughout almost all aquatic environments, which have diverse oxygen concentrations, water pressures, temperatures, and salinities. Related representatives from the same group can be found in different ecosystems. For instance, Perciformes are adapted to both freshwater and marine habitats, including Antarctic. These diverse milieus certainly host a broad variety of pathogens. Fish can be infected by viruses (rhabdoviruses, bornaviruses, reoviruses, nodaviruses, iridoviruses, herpesviruses, etc.), bacteria (*Vibrio*, *Aeromonas*, *Flavobacterium*, *Yersinia*, *Lactococcus*, *Mycobacterium*, etc.), and many parasites. Thus, it is expected that a considerable diversity of host/pathogen interactions characterize fish immune defense mechanisms.

Most of our current knowledge on the immune systems and pathogens of fish comes from aquaculture species. In this context, pathogen diagnostic and vaccination are of considerable economic importance. As an illustration of this, the vaccination program established in Norway to protect Atlantic salmon against vibriosis and furunculosis during the last decades has dramatically reduced the impact of these pathogens, yielding a sharp increase in salmon production that now allows an export value of more than 35 billions Norwegian Kroner (close to 5 billions €) per year. The main aquaculture species of interest for immunology are rainbow trout and Atlantic salmon (*Salmo salar*, Salmoniformes), common, and crucian carp (*Cyprinus carpio* and *Carassius auratus*, Cypriniformes), channel catfish (*Ictalurus punctatus*, Siluriformes), tilapia, sea bass, and sea bream (*Oreochromis niloticus*, *Dicentrarchus labrax*, and *Sparus aurata*, Perciformes), Japanese flounder (*Paralichthys olivaceus*, Pleuronectiformes), as well as cod (*Gadus morhua*, Gadiformes). The immune systems of several additional species of economical importance in Asia like Grass carp (*Ctenopharyngodon idella*, Cypriniformes), and mandarin fish (*Siniperca chuatsi*, Perciformes) have been increasingly studied during the last years. In addition, a few freshwater fish species originally studied in developmental biology for their capacity to provide eggs, or for their ecological/morphological characteristics, later became experimental models in Immunology. These include zebrafish (*Danio rerio*, Cypriniformes), medaka (*Oryzias latipes*, Beloniformes/Cyprinodontiformes), and stickleback (*Gasterosteus aculeatus*, Gasterosteiformes). In sum, it stands out that our knowledge of fish immunology relates only to a minor fraction of the 40,000 known fish species. It is therefore important not to generalize observations made in individual groups, especially since our knowledge on the model species listed above already illustrates that the organization of the immune system differs among distinct fish species.

Besides its direct relevance for aquaculture, the study of the immune system of fish is also of interest to understand the evolution of the adaptive immune system in Vertebrates. The primordial adaptive immune system of extinct vertebrates is not accessible, but it can be inferred through comparative analyses of the B and T cell systems from distant living groups like fish and mammals. Although fish lack bone marrow and lymph nodes, fish infections by bacterial or viral pathogens can lead to the production of specific antibodies, which in some cases correlates perfectly with protection against re-infection by these pathogens. Such a protection may persist for more than 1 year. It is therefore possible to compare how the humoral immune system functions in fish and in mammals.

**Figure 1:**
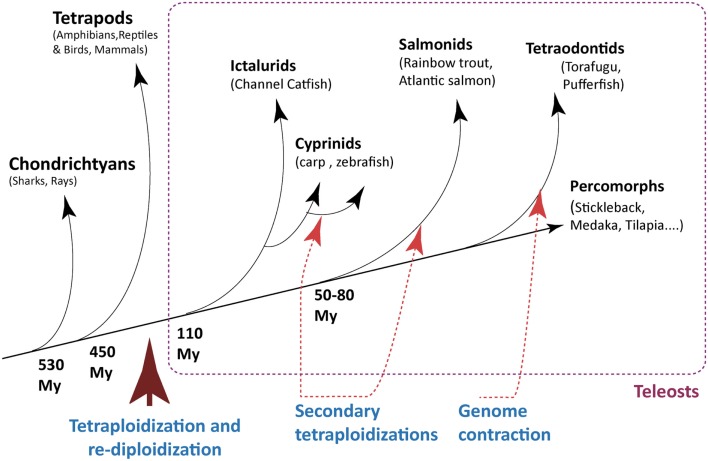
**Milestones of genome evolution within the fish lineage**. A few key events of tetraploidization/re-diploidization and contraction are represented. Note that red arrows indicate a segment on the tree where an event is assumed, not a precise time point. The time arrow is not on scale.

Research on the immune system of fish has generally been limited by the lack of reagents suitable for classical cellular immunology research, but it has greatly benefited from the sequencing of their genomes (Table 1), which have particular structural features directly relevant for their immune system. In particular, a cycle of tetraploidization and re-diploidization occurred during the early evolution of fish genomes, which was followed by further cycles of whole-genome duplications, and differential loss of various genome parts during the subsequent evolution of many fish families (Figure 1). As a result, fish genomes are especially heterogeneous. Some genes involved in the immune system have been affected by these re-modelings; in fact, the great number of gene duplicates has probably played an important role in the diversification of the immune genes through sub-functionalization and specific adaptations. This might also account for the fact that the immunoglobulin (Ig) loci of some fish species are among the largest and most complex described yet. Salmonids have two IgH loci per haplotype with several hundreds of V genes, while mammals have only one IgH loci per haplotype and fewer VH genes.

**Table 1:** **Status of genome sequencing of the main model species for fish immunology**.

**AQUACULTURE SPECIES**	
Rainbow trout (*Oncorhynchus mykiss*)	Genome in progress
Atlantic salmon (*Salmo salar*)	Genome in progress
Atlantic cod (*Gadus morhua*)	Genome published (Star et al., 2011)
Common carp (*Cyprinus carpio*)	Genome published (Henkel et al., 2012)
Crucian carp (*Carassius carassius*)	
Channel catfish (*Ictalurus punctatus*)	Genome in progress
Tilapia (*Oreochromis niloticus*)	Genome available at http://www.ensembl.org/Oreochromis_niloticus/Info/Index
Sea bass (*Dicentrarchus labrax*)	Genome in progress
Sea bream (*Sparus aurata*)	
Japanese flounder (*Paralichthys olivaceus*)	
**MODEL SPECIES**	
Zebrafish (*Danio rerio*)	Genome available at http://www.ensembl.org/Danio_rerio/Info/Index
Medaka (*Oryzias latipes*)	Genome published (Kasahara et al., 2007)
Three-spined stickleback (*Gasterosteus aculeatus*)	Genome published (Jones et al., 2012)

The availability of genomic resources has been particularly useful to investigate B cell repertoires in fish, both for the description of the genomic organization of Ig loci, which defines the potential repertoire, and for the characterization of the primary repertoire expressed by B cells in healthy and infected fish (Jerne, 1971). When considering the importance of efficient adaptive immune responses for the control of infectious diseases, and for successful vaccination, one realizes the relevance of understanding how lymphocyte repertoires are selected during B cell development and modified upon antigenic challenge. In this review, we will first examine fish Ig classes, the structure of the loci, and the IgH splicing patterns. We will then study the B cell system and the features of the available (expressed) repertoires of antibodies in healthy or infected fish.

## Diversification of Ig Genes in Fish: Potential Repertoires and Diversification Mechanisms

### Ig loci in fish

#### Fish have three Ig classes

Three classes of Ig have been identified in teleost fish. These are IgM, which is found in all vertebrate species (reviewed in Flajnik and Kasahara, 2009), IgD, which also has a wide distribution among vertebrates, and IgT/Z (for Teleost/Zebrafish), which is specific to fish. Hereafter, fish IgM, D, and T/Z classes refer to the protein products of the isotypes μ, δ, and τ/ζ, respectively, which correspond to their associated constant genes.

IgM was the first Ig class identified in fish. It can be expressed at the surface of B cells or secreted. Secreted tetrameric IgM represents the main serum Ig in fish.

IgD was initially thought to be expressed only in rodents and primates, and to be of recent evolutionary origin. However, the first fish IgD was identified in Wilson et al. (1997) in the channel catfish. It differs from mammalian IgD because it is a chimeric protein containing a Cμ1 domain followed by a number of Cδ. This chimeric structure was also found in Atlantic salmon (Hordvik et al., 1999), and other fish species (Stenvik and Jørgensen, 2000; Aparicio et al., 2002; Hordvik, 2002; Srisapoome et al., 2004; Xiao et al., 2010). To date, no complete fish IgD heavy chain without Cμ1 has been described. Intriguingly, a similar Cμ1–Cδ structure has been discovered in some non-fish species of the order of the Artiodactyls (Zhao et al., 2002, 2003). Fish IgD also differs from eutherian IgD by the large number (7–17) of Cδ domains it can contain, and by the absence of a hinge. Secreted IgD have been found in catfish (Edholm et al., 2010), and in rainbow trout (Ramirez-Gomez et al., 2012), but with some differences because it did not contain V domain in the former, while it did in rainbow trout. Of note, IgD has been found in most vertebrates, and it has orthologs even in Chondrichthyans (known as IgW), suggesting that it represents a primordial Ig class, like IgM (Ohta and Flajnik, 2006). To date, IgD seems to be missing only in birds, and in few mammalian species. No IgD sequence was found in the chicken *IgH* locus (Zhao et al., 2000) and seems to be absent from the chicken genome. *IgD* could not be found from available sequences from duck and ostrich either (Lundqvist et al., 2001; Huang et al., 2012). In the same line, *IgD* is apparently absent from the elephant and opossum IgH loci (Wang et al., 2009; Guo et al., 2011).

IgT/IgZ was discovered in Hansen et al. (2005) in rainbow trout (IgT) and zebrafish (IgZ; Danilova et al., 2005). It does not exist in other vertebrates but fish. IgHτ/ζ may contain different numbers of C domains: four C domains are found in most species (Salinas et al., 2011), whereas stickleback (*G. aculeatus*) has three and fugu (*Takifugu rubripes*) has two. In carp (*C. carpio*) IgT is a chimeric protein containing a Cμ1 domain and a Cτ/ζ domain (Savan et al., 2005). No Igτ/ζ locus could be found in the Medaka genome or in the Channel catfish, but it might be identified in catfish when the full genome sequence will be available. Recent studies performed in trout demonstrate that IgT is especially critical for the protection of mucosal territories in this species (Zhang et al., 2010), as suggested by the fact that the local ratio of IgT to IgM is >60-fold higher in the gut mucus than in serum. Furthermore, fish surviving an infection by the gut parasite *Ceratomyxa shasta* had elevated titers of parasite-specific IgT only in the gut mucus but not in the serum, while high titers of parasite-specific IgM were measured in the serum but generally not in the mucus. Additionally, as for IgA in human, an important property of IgT in the gut of rainbow trout seems to be its ability to recognize and coat a large percentage of luminal bacteria at steady state. Secreted IgT is found in trout serum as a monomer, and in mucus as a tetramer (Zhang et al., 2010).

Remarkably, neither IgG nor IgE are present in fish, even though long-lasting protection against secondary infection exists, and many parasites can infect fish.

#### Fish IgH loci: structure and number across fish species

**Figure 2:**
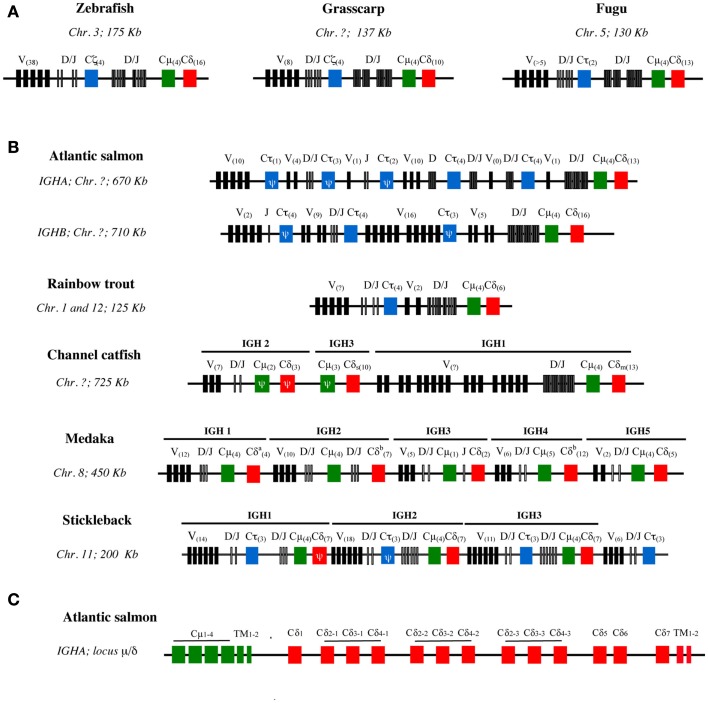
**Schematic structure of IgH loci in different teleost species**. **(A)** IgH loci with archetypic structure in zebrafish, grass carp, and fugu. **(B)** Variants of IgH structure found in other species with partial or complete duplications present in different chromosomes (Chr.) (Atlantic salmon, rainbow trout) or in the same chromosome (channel catfish, three-spined stickleback, and Japanese medaka) (Chr.). The schemes are not in scale and depict the genomic configuration of V sets (black boxes), D and J sets (narrow gray boxes), and CH gene sets. Cμ are represented as green boxes, Cδ as red boxes, and Cτ/ζ as blue boxes. The number of in frame V genes and CH exons are indicated in brackets within boxes. CH sequences with frameshift mutations are considered as pseudogenes (Ψ). Catfish IgH: Cδ_s_ and Cδ_m_ correspond to the secreted and membrane IgD coding genes, respectively. Medaka IgH: in the Cδ^a^, the genomic sequence presents a gap and the actual number of Cδ domains is unknown; Cδ^b^ indicates the presence of Cμ domains inserted between Cδ exons. The “?” symbol indicates a lack of data. **(C)** Detailed exon structure of the IgHA μ−δ region in Atlantic salmon.

The archetypal structure of the IgH loci follows a pattern of translocon organization with a region containing VH genes in 5′, followed by units comprising several D, J, and then C region genes in 3′. The Dτ-Jτ-Cτ cluster(s) encoding IgT specific genes are generally located between the region containing the VH genes and the Dμ/δ-Jμ/δ-Cμ-Cδ locus. This structure is found for example in the zebrafish, grass carp, and fugu (Figure 2A). In this case, the configuration of IgH loci imposes the alternative production of either IgT or IgM/D rearrangements at a given locus since the recombination of VH to Dμ deletes the Dτ-Jτ-Cτ region(s). Since most VH genes are located upstream of both DHτ and Dμ/δ, they can probably be used by IgT, IgM, and IgD (Danilova et al., 2005; Hansen et al., 2005). A large number of VH genes are either pseudogenes, or their sequence is not complete in the genome assembly. Therefore, the diversity of functional VH genes is difficult to estimate. Beyond these general features, the structure of the loci coding for the isotypes corresponding to IgM, IgD, and IgT is surprisingly diverse among teleost fish species, due to successive episodes of genome duplications and gene loss.

##### Various number of IgH loci can be found in teleost species

The number of IgH loci varies among teleosts, and in some cases isoloci can even be found on different chromosomes (Figure 2B).

Salmonids such as Atlantic Salmon and rainbow trout possess two IgH isoloci (IgHA and IgHB) due to the tetraploidization of Salmonidae (Yasuike et al., 2010). The two corresponding IgM subtypes seem to be expressed at the mRNA level in Atlantic salmon and brown trout, but only one is found in rainbow trout and arctic char, suggesting that one of the two isoloci may be non-functional in these last two species. In Atlantic salmon, considering both IgHA and IgHB isoloci, there are eight Cτ loci with variable numbers of Dτ and Jτ genes likely due to tandem duplications, but only three out of these eight loci seem to be functional (two for IgHA and one for IgHB). In contrast, there is only one Dμ/δ-Jμ/δ-Cμ-Cδ region per isolocus.

Cyprinids can also have different types of IgH loci. Zebrafish has only one IgH locus with the archetypic structure, as mentioned above (Danilova et al., 2005). The common carp has two subclasses of IgT/Z: IgZ1 is similar to the zebrafish IgZ while the IgZ2 contains a Cμ1 domain (Ryo et al., 2010). It seems that the two carp IgZ are expressed from two distinct loci, but it is not clear at present whether these loci are located on the same chromosome. The common carp genome has been recently sequenced, and may provide novel information when fully annotated (Henkel et al., 2012).

In other species like channel catfish, medaka, and three-spined stickleback, tandem duplications of the IgH locus have been found (Figure 2B). The channel catfish IgH region contains three μ/δ loci, yet only 1 μ is functional and τ/ζ has not been found so far. The absence of IgT, which still has to be confirmed by full genome sequencing, might be due to a gene loss in the early evolution of Ictalurids. Intriguingly, in catfish the membrane IgD and the (V-less) secreted IgD are always produced from the two different functional Cδ (Bengtén et al., 2006). It remains to be determined whether they could be expressed from the same haplotype. In the medaka genome, five regions encoding constant domains of IgM and IgD have been identified in one large locus (Magadán-Mompó et al., 2011). The analysis of Expressed Sequence Tags (ESTs) suggests that the IGH3 region is disorganized and might be non-functional (Figure 2B). No IgT gene has been found so far in this species. In the stickleback genome, three sets of τ/ζ−μ−δ loci separated by VH-containing regions have been described, evoking recombination units as found in mouse λ light chains or shark IgH loci (Bao et al., 2010; Gambón-Deza et al., 2010).

##### The structure of the IgHδ locus differs between fish species

**Figure 3:**
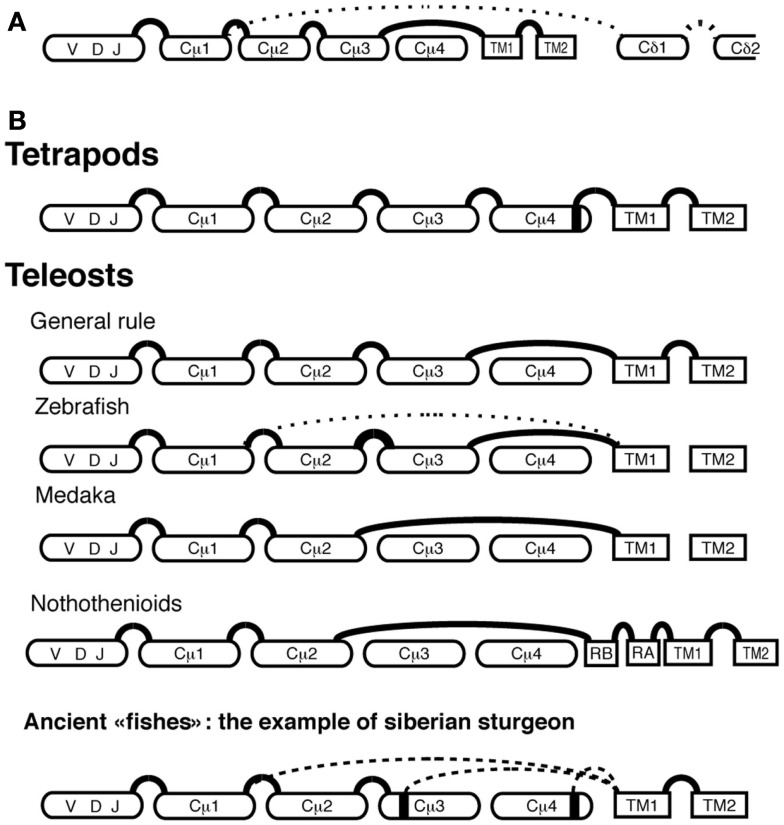
**Representation of IgH splicing alternative pathways in fish and tetrapods**. The alternative splicing leading to IgHμ (plain line) and to IgHδ (dotted line) mature mRNAs **(A)**. IgHμ RNA splicing pathways in different fish groups and in Tetrapods **(B)**: plain and dotted lines represent general and alternative splicing pathways, respectively.

A precise examination of fish IgH shows that the structure of IGHδ is remarkably heterogeneous among fish species with frequent C-domain duplications, while IgHμ and likely IgHτ appear to be more conserved. For example, Cδ2–Cδ3–Cδ4 domains are repeated three times in Atlantic salmon IgHA (Figure 2C) and catfish, and four times in zebrafish and Atlantic salmon IgHB. In puffer fish, the IgD gene comprises a longer tandem Cδ1 → Cδ6 duplication (Saha et al., 2004). The rainbow trout IgD gene is also particular as it carries a Cδ1–Cδ2a–Cδ3a–Cδ4a–Cδ2b–Cδ7 configuration, which seems to be the result of a first duplication of Cδ2–Cδ4 present in Cδ1–Cδ2–Cδ3–Cδ4–Cδ5–Cδ6–Cδ7, leading to Cδ1–Cδ2a–Cδ3a–Cδ4a–Cδ2b–Cδ3b–Cδ4b–Cδ5–Cδ6–Cδ7, followed by deletion of the Cδ3b–Cδ6 domains (Hansen et al., 2005). In the Japanese flounder and stickleback there is no Cδ domain duplication (Hirono et al., 2003; Hansen et al., 2005; Bao et al., 2010; Gambón-Deza et al., 2010). Of note, fish IgM and IgD are co-produced through alternative splicing of a long pre-mRNA containing the VDJ region, the Cμ exons, and the Cδ exons, as in mammals (Figure 3A). Precisely, fish IgHδ mature transcripts are produced by splicing of the donor site at the end of the Cμ1 exon to the acceptor site of the first Cδ exon (Wilson et al., 1997), which results in a chimeric Cμ1/Cδ molecules.

#### Different Ig splicing patterns are used by distinct fish species to generate membrane IgM

In mice and humans, membrane, and secreted IgM H chains are produced from the same pre-mRNA through alternative splicing. A membrane Igμ transcript is made if a cryptic splice site located within Cμ4 is spliced to the acceptor site of the transmembrane (TM)1 exon, and a secreted Igμ transcript is produced when the mRNA is polyadenylated between the last constant (C) region domain Cμ4 and the TM exons. In fish, membrane Igμ transcripts have the TM exons spliced to the donor site located at the 3′end of the Cμ3 exon, hence they lack the last Cμ domain (Cμ4) that is present in the secreted Igμ transcripts (Figure 3B; Bengtén et al., 1991; Lee et al., 1993; van Ginkel et al., 1994). Exceptions to this rule have been found in different species. The medaka membrane Igμ lacks both Cμ3 and Cμ4 domains because the TM exons are spliced directly to the 3′end of the Cμ2 domain (Magadán-Mompó et al., 2011). In the Antarctic Notothenioids fish, membrane Igμ transcripts also lack these domains (Coscia et al., 2010) but two exons consisting of 39-nt (RA and RB) are present between the Cμ2 and TM1 exons (Coscia et al., 2010). This splicing pattern, which is found in most of the Antarctic Notothenioids, may represent an adaptive selection of IgM during Notothenioid evolution (Coscia and Oreste, 2003). In the zebrafish, in addition to the classical VDJ–Cμ1–Cμ2–Cμ3–TM1–TM2 mRNA, an alternative VDJ–Cμ1–TM1–TM2 membrane Igμ transcript has been reported, which encodes only one CH domain (Hu et al., 2011). This implies that B cells can express two different forms of membrane IgM in this species, which increases the number of expressed Ig isotypes. Noteworthy, the functional implication of these various splicing patterns for B cell functions is unknown.

In ancient lineages of fish such as holosteans, the bowfin (*Amia calva*), and the long-nose gar (*Lepisosteus osseus*) a remarkable diversity of splicing patterns of the membrane Igμ transcripts was also observed (Wilson et al., 1995a,b). In chondrosteans, another ancient fish lineage, the diversity of membrane Igμ transcripts is even higher: in Siberian sturgeon (*Acipenser baerii*) the TM1 exon is alternatively spliced to three possible donor sites: a cryptic site at the end of Cμ4, a cryptic site at the end of Cμ3, and the donor splice site at the 3′end of Cμ1, leading to IgM H chains with four, two, or only one complete Cμ domain(s) (Lundqvist et al., 2009; Figure 3B). The shortest membrane Igμ splice variant might have specialized functions because it was retrieved only in transcripts expressing VH2 (Lundqvist et al., 2009). This diversification of splicing pathways to produce membrane IgM in the “ancient fish” lineages evokes a highly diverse situation after whole-genome duplication in the early fish evolution, followed by standardization to the Cμ3 → TM1 splicing pattern in teleosts before their great radiation. However, the particular situations found in ice fish (Notothenioids) or even in zebrafish or medaka indicate that this standardization is not universal.

#### IgL loci in teleost fish

Four immunoglobulin light chain (IgL) isotypes have been described in teleosts: L1, L2, L3, and λ (Edholm et al., 2009; Bao et al., 2010). A recent comprehensive phylogenetic analysis of vertebrate VL and CL sequences suggested that fish L1 and L3 chains are κ orthologous (Criscitiello and Flajnik, 2007), and fish L2 are orthologs of Xenopus σ (Partula et al., 1996). The current general classification of IgL from all vertebrates distinguishes four clans based on phylogenetic relationships: κ (mammalian κ, elasmobranch Type III, Teleost L1, and L3, Xenopus ρ), λ (mammalian λ, elasmobranch type II), σ (Xenopus σ, teleost L2, elasmobranch type IV), and σ cart (σ-cart, that is restricted to elasmobranch).

Light chain genes in fish genomes are found as multiple VL–JL–CL units. The genomic organization of VL–JL–CL unit is conserved in teleosts. For the L1 and L3 loci, the V genes are in opposite transcriptional orientation with respect to the J and C segments. In contrast, in L2 and λ clusters V, J, and C genes are in same orientation (Daggfeldt et al., 1993; Ghaffari and Lobb, 1993, 1997; Timmusk et al., 2000), with the exception of stickleback L2 (Bao et al., 2010). In a genome wide study in zebrafish, such clusters have been found in five different chromosomes (Zimmerman et al., 2008). Interestingly, VL–JL rearrangements between distinct units were reported in this species, which might be a means of increasing the potential combinatorial diversity.

It is intriguing that to date no pseudo light chain corresponding to eutherian mammal VpreB or λ5 has been reported in fish. In mammals, these chains play a crucial role in the stepwise process of Ig chains rearrangements that take place during B cell development. A deficiency in VpreB or λ5 results in a block of B cell development at the pre-B cell stage in mice (Kitamura et al., 1992). In fish, it is still unknown if an alternative pre-B cell receptor that lacks the VpreB/λ5 surrogate light chain forms during B cell development. In fact, it is not known if Ig gene rearrangements in fish follow the ordered model described in mouse and human; moreover, no pre-Tα receptor has been found in fish, while it was recently discovered in sauropsids (Smelty et al., 2010). Similarly, the mechanisms ensuring allelic exclusion in fish are unknown.

### Pathways and enzymatic machinery of Ig rearrangement and diversification

#### The enzymatic machinery of Ig gene rearrangement: similarities between fish and mammals

The rearrangement of VDJ genes is mediated in mammals by a complex enzymatic machinery that includes recombination activating genes (RAG)-1 and 2, proteins from the non-homologous end joining (NHEJ) pathway of repair of DNA double strand breaks, and DNA polymerases of the X family polymerase λ, polymerase μ, and terminal deoxynucleotidyl transferase (TdT). RAG are lymphocyte-specific enzymes that mediate the first steps of VDJ recombination including recognition of the Recognition Sequence Signal (RSS) situated on the sides of the Ig gene segments recruited in the rearrangement, cleavage of DNA at these RSS sites, and hairpin formation as well as resolution. The NHEJ components (Ku70, Ku80, DNAPK, XRCC4, ligase IV, and ARTEMIS) constitute the major pathway involved in the repair of the double strand DNA breaks introduced by the RAG enzymes. The resolution of the DNA breaks is preceded by the action of polymerases λ and μ, which mediate DNA deletional trimming at the junction site, and TdT, which adds “N” nucleotides in a template-independent manner in VDJ junctions. The enzymes implicated in the molecular machinery of Ig rearrangements are remarkably conserved between mammals and fish (Table 2).

**Table 2:** **Genes of the key participants of the rearrangement machinery in fish**.

	Reference	Zebrafish	Stickleback
*Rag1*	Hansen and Kaattari (1995), Hansen and Kaattari (1996), Greenhalgh and Steiner (1995), Willett et al. (1997)	ENSDARG00000052122	ENSGACG00000011465
*Rag2*		ENSDAzRG00000052121	ENSGACG00000011461
**NHEJ**			
*Ku70*	Bladen et al. (2007)	ENSDARG00000090718	ENSGACG00000004868
		ENSDARG00000071551	
*Ku80*		ENSDARG00000068862	ENSGACG00000006130
		ENSDARG00000015599	
*XRCC4*		ENSDARG00000010732	? (But present in a number of other fish)
*DNAPK*		ENSDARG00000075083	ENSGACG00000001974
*Artemis/DCLRE1C*		ENSDARG00000045704	ENSGACG00000020073
*Ligase IV*		ENSDARG00000060620	ENSGACG00000014135
**POLYMERASES X**			
*Polymerases* λ		ENSDARG00000039613	ENSGACG00000018272
*Polymerases* μ	Beetz et al. (2007)	ENSDARG00000042507	ENSGACG00000001887
*TdT*	Hansen (1997), Beetz et al. (2007)	ENSDARG00000038540	ENSGACG00000002880

RAG1 and RAG2 from fish were first cloned in rainbow trout (Hansen and Kaattari, 1996; Hansen, 1997) and zebrafish (Greenhalgh and Steiner, 1995; Willett et al., 1997). They are expressed in tissues where rearrangement activity is expected, and a zebrafish with a truncated RAG1, identified by screening of *N*-ethyl-*N*-nitrosourea mutants, is unable to make VDJ rearrangements, indicating that this enzyme is required for this process in zebrafish as in mammals (Wienholds et al., 2002). In line with this, V, D, and J segments of fish IgH and IgL are flanked by typical RSS (Ghaffari and Lobb, 1997; Hayman and Lobb, 2000). Of note, RSS-like heptamers and nonamers were found within some JL–CL introns (Ghaffari and Lobb, 1997) as well as in 3’ region of the majority of the zebrafish CL genes (Zimmerman et al., 2011), evoking the isolated RSS heptamer recombination element located in mouse Jκ–Cκ intron, which can recombine with the κ-deleting element located downstream of Cκ exon to delete the Cκ exon and silence the Igκ locus (Vela et al., 2008). Such process of locus inactivation might provide a mechanism to achieve allelic exclusion for fish IgL (Vela et al., 2008).

The genes coding for the main enzymes of the NHEJ machinery appear to be present in fish genomes, with (recent) duplications for some of them in zebrafish (Table 2). An ortholog of Ku70 was identified in zebrafish that was critical for protection from radiation-induced DNA damage because embryos in which this gene was knocked-down were highly sensitive to ionizing radiation (Bladen et al., 2007).

Orthologs of the X family of DNA polymerases involved in diversification of VDJ junctions have also been identified in fish. The gene coding for TdT was found in rainbow trout and zebrafish genomes (Hansen, 1997; Beetz et al., 2007). It is expressed in lymphoid tissues where rearrangements occur (thymus, pronephros, mesonephros, spleen, and gut). Both TCR and Ig junctions contain N diversity, suggesting that fish TdT has similar functions as in mammals. In zebrafish polymerase μ is expressed also in primary lymphoid tissues, as well as in ovary and testis (Beetz et al., 2007). Thus, the mechanisms of Ig rearrangement might be similar in teleosts and mammals.

It is interesting to note that the genes coding for some of these enzymes are present in the genomes of ancient fish such as cartilaginous elasmobranch (which include shark, ray, and skates). TdT from elasmobranch has structural similarities with the mouse TdT, in agreement with the fact that both enzymes have template-independent mode of DNA elongation without strong nucleotide bias (Bartl et al., 2003). These data suggest that TdT and other polymerases from the ancient family of polymerases X were used by the rearrangement machinery even before the divergence of fish and mammals (Beetz et al., 2007).

#### Mechanisms of hypermutation: presence and limits

The affinity maturation of antibody responses is less efficient in cold blood vertebrates compared to mammals (Wilson et al., 1992). For example after immunization of rainbow trout with the hapten-carrier antigen TNP-KLH (trinitrophenyl-linked to keyhole limpet hemocyanin), the affinity of the antigen-specific antibody response progressively increased over 27 weeks, with initial production of low affinity antibodies, which were replaced within 5 weeks by antibodies of intermediate affinity, and after 15 weeks by antibodies that had the highest affinity for antigen (Ye et al., 2011). It is assumed that the low efficiency of the affinity maturation of the antigen-specific antibody response in fish is due to the absence of typical germinal centers (GC), which are the specialized anatomical structures supporting the selection of B cells expressing high affinity B cell receptor (BCR) for antigen in mammals (Wilson et al., 1992). However, clusters of cells containing melano-macrophages were found in spleen and kidney of channel catfish, which might represent primordial GC because activation-induced deaminase (AID) was expressed in these structures (Saunders et al., 2010). AID is a critical enzyme for somatic hypermutation and class switch recombination of Ig genes in mammals. Fish AID differ from their mammalian counterparts at the level of the catalytic sites, but puffer fish and zebrafish AID could nonetheless mediate Ig class switch recombination in mouse B cells (Barreto et al., 2005). In catfish hypermutated IgH sites show an accumulation of G → A and C → T substitutions consistent with AID activity. However, the pattern of Ig somatic hypermutation has particular characteristics in fish, with sequence motifs containing hypermutation hotspots different from those known in mammals (Yang et al., 2006). Interestingly, there was no difference in the ratio of replacement-to-silent mutations in the complementarity determining regions (CDR), which correspond to the Ig parts involved in antigen binding, and in the framework regions, which are normally not involved in antigen binding. Thus, the mechanism of Ig somatic mutation did not coevolve in fish with the pathways mediating selection of B cells with non-synonymous substitutions specifically within CDR-encoded regions. Fish Ig structure suggests that as in mammals CDR are most important for antigen binding, and that they form the main part of the antigen binding surface. A possible explanation for this finding is that mutated Ig sequences do not undergo positive selection for affinity maturation efficiently due to the lack of an appropriate micro-environment. In this context, the primary role of the process of somatic hypermutation might have been to diversify the available repertoire by targeting hotspot motifs preferentially located within CDR-encoded regions. Whether part of this diversity might have deleterious specificity and require particular negative selection remains unknown. In zebrafish, a comprehensive analysis of IgHμ transcripts via deep sequencing indicated that the frequency of Ig sequences with high numbers of somatic mutations increased with age (up through 1 year), in agreement with the notion that hypermutation brings a significant contribution to the diversification of the Ig repertoire (Jiang et al., 2011). Fish Ig light chains can also be subjected to hypermutation (Marianes and Zimmerman, 2011), as previously observed in shark (Lee et al., 2002). It is so far unknown whether fish AID, like mammalian ones, can specifically target additional genes with frequent translocations in tumors, repetitive sequences, and histone H3K4 trimethylation (Kato et al., 2011). The gene *aid* is found in the genome of the main fish model species within conserved synteny groups, indicating they represent true orthologs of the mammalian gene (see zebrafish ENSDARG00000015734, stickleback ENSGACG00000010521, fugu ENSTRUG00000007079 in the Ensembl website).

### The central B cell system in fish

Three modes of early hematopoiesis have been described in fish (Zapata et al., 2006): hematopoiesis can start in the yolk sac blood islands like in the angelfish, or in intraembryonic intermediate cell mass (ICM) as in zebrafish; alternatively it may initiate for a short time in the yolk sac before continuing in the ICM as in rainbow trout. In zebrafish, the hematopoietic activity appears at 4 days post-fertilization (dpf), but gives rise first to erythroblasts and myeloid cells. Fish B cell lymphopoiesis appears and occurs mainly in the kidney. The expression of zebrafish RAG2 was observed at 8 dpf in the pronephros of *Rag2*-*Gfp* transgenic fish, which was the earliest extrathymic site of RAG expression (Trede et al., 2004). AID mRNA was even detected at 2 dpf in this species by analysis of gene expression on the whole embryo (Trede et al., 2004). The first VHDHJH rearrangements were detected around 4 dpf (Danilova and Steiner, 2002), but cells expressing IgM (Lam et al., 2004) appeared in the kidney only at 3 weeks post-fertilization, suggesting a slow process of B cell maturation. In the rainbow trout, RAG expression occurred earlier, from 10 dpf onward, and membrane IgM-expressing cells became detectable at hatching (Razquin et al., 1990), around 3 wpf (Hansen, 1997). The spleen seems to have much less importance for B cell lymphopoiesis than the kidney tissue, if any.

In adult fish, B cells reside in the anterior and posterior kidney, spleen, gut lamina propria, and blood (Rombout et al., 1993; Abelli et al., 1997). Several B cell subsets can be distinguished according to their expression of distinct Ig class combinations. In some fish species two subsets of B cells can be identified by their expression of both IgM and D, or IgT only. The development of IgM^+^IgD^+^ B cells and IgT^+^ B cells involves two different pathways because in zebrafish with a deficiency in *Ikaros* gene IgT^+^ B cells are totally lacking, while IgM^+^ B cells are present, even though their appearance shows a delayed kinetic (Schorpp et al., 2006). Moreover, these two types of B cells are differently localized in the organism. IgM^+^ B cells are the main B cell population (75–80%) in spleen, kidney, and blood, while IgT^+^ B cells represent the main B cell subset (55%) in gut-associated lymphoid tissues (Zhang et al., 2010). The existence and importance of IgM^−^IgD^+^IgT^−^ B cells in fish is a matter of debate. While in most fish species it is considered that IgD is always co-expressed with IgM, a distinct population of IgM^−^IgD^+^ B cells has recently been identified in the channel catfish, which preferentially expresses σ IgL (Edholm et al., 2010). The frequency of this population is highly variable between individuals, ranging from a few percent to more than 70% of B cells within peripheral blood leukocytes. The participation of these cells to immune responses is not known.

Fish B cells show different homing patterns depending on their development and activation stages. B cell progenitors and plasma cells are dominant in the anterior kidney, while mature B cells and plasma blasts are primarily found in posterior kidney (Zwollo et al., 2005; Zwollo, 2011). Spleen leukocytes also contain B cells that can differentiate into plasma cells. Based on these data, it can be envisioned that B cell development occurs in the anterior kidney, from where mature B cells enter the blood/lymph to reach the spleen and posterior kidney, where they can become activated and differentiate into plasma blasts and then plasma cells, which migrate back to the anterior kidney where they might subsist as long-lived cells in particular niches. Such model suggests that B cells use the same tissue for their development as plasma cells for their residence, as previously observed in mammals.

#### The B cell repertoire in the healthy fish

The modalities of B cell selection to produce a naïve repertoire remain unknown in fish. The development of high-throughput sequencing methods now makes possible a comprehensive description of expressed immune repertoires. The first exhaustive sequencing of a B cell expressed diversity in a vertebrate was performed in zebrafish by Weinstein et al. (2009) using 454 GS FLX pyrosequencing. In this study, whole-fish mRNA was prepared from 14 individuals belonging to 4 families, and the variable domain (VDJ region) of IgHμ sequenced. The expressed IgM repertoire was studied in quiescent state, from healthy fish that had been raised in classical aquarium environment and possessed a normal gut microbial flora. It was estimated that a large proportion of the possible V/J combinations (50–86%) were expressed. Interestingly, the distribution of VDJ diversity was similar between individuals, and identical μ heavy chains were found in distinct fish more often than expected. This study established that the expressed IgM repertoire of different fish belonging to distinct families shared some patterns, a property which was called stereotypy. The same laboratory also followed the evolution of the expressed IgM repertoire during zebrafish development (Jiang et al., 2011). In 2-weeks-old fish, the repertoire of VDJ combinations showed a high level of stereotypy, suggesting that the primary repertoire was strongly constrained. In such young fish, which have few (if any) antibody-secreting cells, the abundance, and the junctional sequence diversity of VDJ combinations correlated. In contrast, this correlation was lost in 3-month-old fish. This was likely due to the higher frequency of antibody-secreting cells in these older animals. Nevertheless, the frequencies of VDJ combinations correlated between individuals, substantiating further the notion that deterministic forces regulate the structure of the primary repertoire. The apparent contradiction between a deterministic view of the expression of VDJ combinations and the loss of correlation between VDJ frequency and diversity in adult fish may be explained by the accumulation of different numbers of plasma cells in distinct adult fish.

In a different study, a combination of CDR3 length spectratyping and pyrosequencing was used to describe the expressed IgM, IgD, and IgT repertoires in rainbow trout (Castro et al., 2013). The VDJ domains expression was studied in the spleen of naïve individuals. Clonal isogenic animals were analyzed to avoid fish-to-fish variation due to genetic heterogeneity. As in zebrafish, it was found that not all V/J combinations were expressed. In fact, only 7 out of 13 VH families were retrieved. CDR3 length spectratyping and pyrosequencing showed that spleen Ig repertoires were very diverse for all three isotypes in healthy fish. IgM and IgD repertoires were rather similar for most VH, while being significantly different from the IgT repertoire. This observation suggested that IgM and IgD repertoires were not subjected to drastic differential selection. The strong difference between IgT and IgM/IgD CDR3 length profiles was consistent with the usage of a different set of rearrangements with specific D and J segments in B cells expressing either IgM/IgD or IgT. A more detailed analysis focused on the VDJ junctions. To compare the distributions of junctional sequences between individuals, sequence reads encoding a CDR3 region were annotated using IMGT/highV-QUEST for VH, JH, and C genes, and aggregated into “junction sequence types” (JST). The abundance distribution of JST computed from pyrosequencing datasets indicated that 90–99% of junction sequences were found less than five times, likely corresponding to naive non-expanded B cells. Only few JST were found more than 20 times, possibly reflecting the presence of few antibody-secreting cells in the spleen of these fish, in good accordance with previous studies about spleen B cell subsets in rainbow trout (Bromage et al., 2004).

Taken together, these observations indicate that all VDJ combinations are not equally expressed, and suggest that, at least in zebrafish, the expressed repertoire exhibits a significant level of stereotypy.

## The Modifications of Fish Expressed Ab Repertoires by Infections and Vaccines

In fish, B cell responses occur against a variety of pathogens, and must occur in microenvironments different from those described in mammals, due to the lack of GC and lymph nodes. In this context, the clonal complexity of trout B cell responses is largely unknown. The development of high-throughput sequencing approaches of Ig transcripts combined with CDR3 length spectratyping can provide comprehensive analyses of B cell responses, which are required to understand the dynamics of their clonal complexity (Ademokun et al., 2011).

Such an approach was used to characterize the B cell response of rainbow trout against a rhabdovirus, the Viral Hemorrhagic Septicemia Virus (VHSV). Clonal fish were vaccinated using an attenuated virus, then challenged 3 weeks later with the same virus, and finally analyzed after three more weeks. At this stage, all fish had neutralizing antibodies against VHSV, and increased levels of total IgM as well as IgT in serum. The titer of IgM remained more than 10 times higher than of IgT after infection, and the ratio of IgM^+^IgT^−^/IgM^−^IgT^+^ B cells was similar between infected and control fish. CDR3 length spectratyping showed that the VHSV infection triggered a strong IgM response. Indeed, VHCμ spectratypes were extensively and significantly skewed in infected fish for all the analyzed VH, as shown by a comparison of each peak in each spectratype profile using the ISEApeaks software (Collette and Six, 2002). Interestingly, the VH5.1-Cμ profile showed a great amplification of the same peak in all infected individuals, suggesting a public response. In contrast, VHCδ profiles showed only weak and sporadic alterations, which were not statistically validated. The low contribution of IgD to the response might reflect a down regulation of its expression in activated B cells, as in human and mice. This analysis also revealed a significant IgT response in spleen of infected fish. After VHSV infection, most splenic IgT spectratyping profiles were affected, although to a lesser extent compared to IgM. No peak expansion common to all infected fish was observed for IgT, suggesting the absence of a public response. Hence, the spleen might be a site of activation for VHSV-specific IgT^+^ B cells. This is intriguing because IgT is a specialized mucosal Ig.

**Figure 4:**
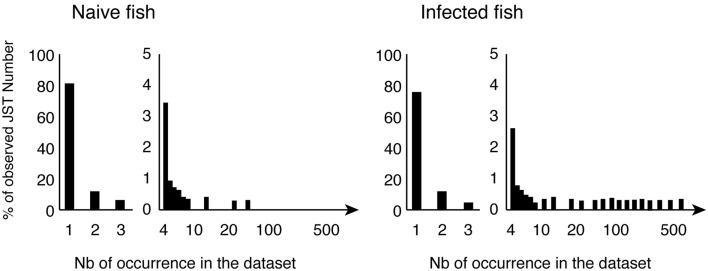
**Typical normalized distributions of JST in the pyrosequencing datasets**. JST observed n times from control and virus infected fish for a given VH/C combination are represented as percentages of the total number of JST. Large clonal expansions are indicated by high number of occurrences of expressed JST in infected animals.

The molecular diversity of IgM and IgT responses was further characterized by pyrosequencing of VHC junctions for different VH groups. Since a JST corresponds to a CDR3 protein sequence associated with a (VH, JH) pair, the distribution of the relative abundance of JST in different fish provides a description of the importance of antibody clonal responses. IgM JST distributions showed that the virus induced a major shift of the IgM expressed repertoire, with appearance of a significant number of highly represented JST (Figure 4).

Further analysis indicated that these large JST sets corresponded essentially to transcripts encoding secreted IgH, hence to antibody-secreting cells. When comparing the JST expanded in different infected fish, similar VH5.1-J5 rearrangements with CDR3 of 10 amino acids were present in all individuals. The CDR3 with the amino acid sequence ARYNNNAFDY was the most frequent, but a number of other related JST were found repeated in several individuals, with exchange of small or polar amino acids: ARYNN**D**AFDY, ARY**D**NNAFDY, ARYN**S**NAFDY, ARYNN**V**AFDY, ARY**DD**NAFDY, ARYN**T**NAFDY, ARYN**GD**AFDY, ARY**SGD**AFDY, and ARYN**GR**AFDY. Such expansion of a number of similar junctions found in several fish, and differing from the most frequent one by only one (or a few) conservative substitution(s) is typical of “public” responses in mammals (Bousso et al., 1998; Lin and Welsh, 1998). Importantly, this observation suggests that rainbow trout possess a common pool of pre-existing spleen VH5.1^+^ B cells among which the public IgM response to VHSV is recruited.

This pyrosequencing study also revealed the great importance and diversity of private clonal expansions in infected fish. It is at present unclear whether these expansions represent only VHSV-specific responses or include bystander-activated cells. It will be important to clarify this point, and whether bystander effects could be beneficial or detrimental to the host. In this regard, it is intriguing that fish injected with oil-adjuvanted vaccines developed an autoimmune syndrome with autoantibodies and liver lesions (Koppang et al., 2008).

## Perspectives

The aim of this review was to provide a concise description of our current knowledge of fish Ig repertoire. It is clear that a lot of important unknowns remain in fish B cell biology. As perspectives, we have listed five topics below, which might provide interesting areas for future investigation.

### B cell receptor allelic exclusion in fish

Many aspects of Ig gene rearrangement and B cell biology remain mysterious in fish. In particular, the absence of the pseudo light chains VpreB and λ5, which are required for the formation of pre-BCR in mammals, suggests that allelic exclusion is achieved by different mechanisms in fish and mammals. In fact, the regulation of VDJ recombination to ensure allelic and isotypic exclusion in fish is far from being understood. This question evokes the situation in sharks where the IgH locus organization consists of many (up to 200) independently rearranging miniloci: in these species, the rearrangement takes place within a minilocus, and only one or few H chain genes are fully rearranged in each B cell, whereas the other loci retain their germline configuration (Malecek et al., 2008). The mechanisms by which sharks and bony fishes regulate the progression of VDJ rearrangements might reveal pathways of general interest.

### Maturation of B cell response without GC

The process of hypermutation of Ig genes observed in fish in absence of typical GC is reminiscent of the affinity maturation that can occur in mammals in extra follicular foci in the spleen red pulp (Matsumoto et al., 1996). Its potential role in the diversification of the fish Ig repertoire also bears some similarities with the fact that at least a part of human marginal zone B cell pool expresses a BCR repertoire diversified through somatic hypermutation independently of GC, even though antigen stimulation via BCR does not seem to be involved in the latter case (Weill et al., 2009). Collectively, these examples highlight the diverse utilizations made during evolution of this remarkable process of somatic hypermutation of Ig genes, for the diversification of antibody repertoires. In fish, the existence of long-term protection and antigen-specific B cell memory raises the question of differentiation of memory B cells in absence of classical GC. In fact, memory B cells expressing high affinity, hypermutated IgG1 were found in lymphotoxin-alpha deficient mice, which lack GC (Matsumoto et al., 1996). The alternative site of memory B cells differentiation has not been identified. The modalities of memory B cell formation outside GC represent both a practical issue for vaccination and a fundamental question in B cell biology.

### Diversity of B cell repertoires

The comprehensive description of fish B cell repertoires and in-depth statistical analyses have opened the way to comparative studies of the population dynamics of B cells in different fish species. The seminal work of Quake’s group suggests that zebrafish antibody repertoires may harbor a higher level of stereotypy than expected. It will be interesting to understand if the total number of B cells present at a given time has a strong influence on such patterns: a zebrafish may contain a few millions of B cells, while a trout has around 100–1000 times more, and a large tuna probably 1000–10,000 times more. It appears likely that the constraints exerted on B cell diversity to express at once a complete repertoire able to cope properly with the diversity of relevant pathogens will be different in these species. Also, some fish species like Atlantic cod show very poor antibody responses (Espelid et al., 1991; Pilström and Petersson, 1991; Schrøder et al., 1992; Magnadottir et al., 2001), when having high level of serum antibodies and a repertoire strongly skewed toward the VHIII family (Stenvik et al., 2001), possibly reflecting a particular importance of natural antibodies. These particularities must be put in the context of the absence of CD4, LI, and MHC class II molecules (hence, lack of the equivalent of a CD4^+^ T cell help activity) recently revealed by the analysis of the complete sequence of the cod genome (Star et al., 2011). As a group, teleost fish represent a rich diversity of species with a wide range of size and a complex history of whole-genome duplications. Future studies on B cell repertoires from different fish species will provide insightful information about the general rules of adaptation of this system, in fish and more generally in vertebrates.

### Methodologies for B cell repertoire analysis

CDR3 length spectratyping, also called Immunoscope, has been the standard technique for large-scale analysis of antigen receptors repertoire diversity for about 15 years (Pannetier et al., 1993, 1995). Systematic sequencing of “all” Ig transcripts expressed in a lymphocyte population of interest represents a step forward, and is made possible by the “next generation” sequencing technologies. A benefit of these approaches is clearly that several angles of analysis can be taken to focus on different aspects of the repertoire such as clonotypes frequency, Ig V-C or V-J CDR3 diversity, CDR3 sequence analysis, V allele identification, etc. The ability to process the complexity of the information provided in such amounts of data remains limited, and specific software developments for automatic annotation of Ig sequences, and statistical modeling of repertoire diversity can still be improved. New strategies will have to be developed, possibly from existing scoring systems. The most common is the Shannon entropy, introduced by Claude Shannon in 1948 for the information theory. Then, in 1961, Alfred Rényi has generalized the utilization of an entropy index to several functions, including Species Richness, Simpson, Quadratic, and Berger–Parker indexes to quantify the diversity, uncertainty, or randomness of a system, respectively. Among these, Simpson’s diversity and Shannon’s entropy indices have already been applied to analyze TCR sequence data. A comparative review of such scoring strategies was published by Miqueu et al. (2007). Deep sequencing repertoire analysis calls for advanced statistical analysis and graphical representations, such as multivariate analysis (e.g., hierarchical clustering, principal component analysis, multidimensional scaling, etc.) and probabilistic or network modeling of sequence distributions (Mora et al., 2010; Ben-Hamo and Efroni, 2011; Murugan et al., 2012). In this perspective, different parameters can be computed to quantify the differences between repertoires at distinct levels. An important feature is the total diversity of the repertoire, which can be estimated from a dataset following approaches (Fisher et al., 1943; Efron and Thisted, 1976). At another level, a deep sequencing dataset can be summarized in various groups of sequences sharing common features (e.g., V or J gene segment, CDR3 length, sample origin, frequency), which allows comparisons between different conditions. For example, a perturbation score can be computed from the Hamming distance (Gorochov et al., 1998) to compare antibody repertoires between infected fish and a reference from control animals.

### Effect of temperature on fish B cell responses

While fish have colonized aquatic environments across a wide temperature range, only a few species control their internal temperature. The adaptation of fish immune system to various temperature is not fully understood, but the magnitude of the primary response to T dependent antigens is suppressed at lower temperatures for example in the channel catfish (Bly and Clem, 1991) and carp (Le Morvan et al., 1996). More recently, it was also observed that the highest magnitude of rainbow trout specific IgM – but not IgT – response against *Yersinia ruckeri* was obtained at high temperature (25°C; Raida and Buchmann, 2007). Differential sensitivity of lymphocyte responses to temperature variations may affect immune repertoires – perhaps especially regarding natural antibodies and mucosal locations – since different pathogens may be adapted to distinct temperature ranges.

With a large number of species and a wide diversity of anatomy, physiological, and ecological adaptations to the aquatic environments and their pathogens, fish offer interesting perspectives for comparative analysis of B cell repertoire biology. New sequencing technologies have already made it possible.

## Conflict of Interest Statement

The authors declare that the research was conducted in the absence of any commercial or financial relationships that could be construed as a potential conflict of interest.
